# ArecaNet: Robust Facial Emotion Recognition via Assembled Residual Enhanced Cross-Attention Networks for Emotion-Aware Human–Computer Interaction

**DOI:** 10.3390/s25237375

**Published:** 2025-12-04

**Authors:** Jaemyung Kim, Gyuho Choi

**Affiliations:** 1 IT Research Institute, Chosun University, Gwangju 61452, Republic of Korea; kjm@chosun.ac.kr; 2 Department of Artificial Intelligence Engineering, Chosun University, Gwangju 61452, Republic of Korea

**Keywords:** facial emotion recognition, multi-model ensemble, attention fusion, residual cross-attention, computer vision

## Abstract

Recently, the convergence of advanced sensor technologies and innovations in artificial intelligence and robotics has highlighted facial emotion recognition (FER) as an essential component of human–computer interaction (HCI). Traditional FER studies based on handcrafted features and shallow machine learning have shown a limited performance, while convolutional neural networks (CNNs) have improved nonlinear emotion pattern analysis but have been constrained by local feature extraction. Vision transformers (ViTs) have addressed this by leveraging global correlations, yet both CNN- and ViT-based single networks often suffer from overfitting, single-network dependency, and information loss in ensemble operations. To overcome these limitations, we propose ArecaNet, an assembled residual enhanced cross-attention network that integrates multiple feature streams without information loss. The framework comprises (i) channel and spatial feature extraction via SCSESResNet, (ii) landmark feature extraction from specialized sub-networks, (iii) iterative fusion through residual enhanced cross-attention, (iv) final emotion classification from the fused representation. Our research introduces a novel approach by integrating pre-trained sub-networks specialized in facial recognition with an attention mechanism and our uniquely designed main network, which is optimized for size reduction and efficient feature extraction. The extracted features are fused through an iterative residual enhanced cross-attention mechanism, which minimizes information loss and preserves complementary representations across networks. This strategy overcomes the limitations of conventional ensemble methods, enabling seamless feature integration and robust recognition. The experimental results show that the proposed ArecaNet achieved accuracies of 97.0% and 97.8% using the public databases, FER-2013 and RAF-DB, which were 4.5% better than the existing state-of-the-art method, PAtt-Lite, for FER-2013 and 2.75% for RAF-DB, and achieved a new state-of-the-art accuracy for each database.

## 1. Introduction

Human–computer interaction (HCI) technologies are increasingly expected to provide personalized and adaptive services in domains such as healthcare, education, and recommendation systems [1]. However, effective personalization requires systems that accurately perceive and respond to users’ emotional states. Without reliable emotion recognition, HCI systems risk reduced trust, limited engagement, and poor decision-making support. In this context, facial emotion recognition (FER) has emerged as a key technology, as facial expressions provide natural, universal, and non-intrusive cues for human affect. Compared with other modalities such as biometric expression recognition (BER) and speech emotion recognition (SER), FER offers real-time accessibility and strong applicability in practical environments, making it indispensable for building intelligent, emotion-aware interactive systems [2].

At the same time, FER mainly depends on camera-based vision sensors, which capture facial images as the primary input. These sensors, however, are vulnerable to practical challenges such as illumination changes, occlusions from masks or glasses, and variations in head pose, all of which can significantly reduce recognition accuracy [3]. In addition, real-world deployment often involves low-resolution data and heterogeneous sensor devices, further complicating performance stability. Recent studies have explored ways to address these challenges in practical scenarios. For example, Lawpanom et al. [4] developed a system that analyzes students’ emotional states to personalize online learning, while Leo et al. [5] proposed a healthcare application that combines FER with BER to monitor patients and design tailored treatment plans. FER is also being extended to applications such as personalized healthcare, education, and recommendation services through integration with other modalities. Nevertheless, existing systems remain sensitive to sensor-induced variability, underscoring the need for FER methods that can operate robustly across diverse sensing conditions to ensure dependable HCI performance.

Islam et al. [6] recognized emotions through feature extraction algorithms such as HOG and LBP and SVM, a representative machine learning model for the initial FER system. Unlike the deep learning network methods, the HOG and LBP feature extraction methods extract handcrafted features by separating the feature extraction step from the classification step; but, the accuracy of emotion classification was verified to be low due to the limitation of learning high-dimensional nonlinear patterns. With the development of deep learning networks, Krizhevsky et al. [7], who used convolutional neural networks (CNNs), verified the effectiveness of high-dimensional nonlinear pattern analysis through filter-based convolution operations. Ravi et al. [8] verified the superiority of a CNN by comparing and analyzing two methods, LBP and CNN, on the same FER database. CNN-based networks extracting local features face limitations due to the inductive bias of local connectivity and spatial invariance. It is difficult to consider the correlation of local features such as eyes and mouths in the feature extraction process of facial images using CNN-based networks. The vision transformer (ViT) proposed by Dosovitskiy et al. [9] solved the limitations of CNNs by obtaining correlation information between each patch through an attention block of a linearly projected image in patch units and using global features of expressions. Chaudhari et al. [10] verified that ViT had higher a accuracy than CNN in FER. CNN and ViT are easily overfitted to the training DB when designed as a single network, making it difficult to improve classification accuracy with high sensitivity in real-world environments. To address the limited performance of a single network, Xu et al. [11] proposed a voting ensemble method, Singh et al. [12] developed a score-level ensemble method, and Karnati et al. [13] proposed a feature-level ensemble method. Existing ensemble networks’ performances are determined by a single network, so important information exchange between the networks is insufficient and the feature information of each network is lost during the ensemble process.

This paper proposes an assembled residual enhanced cross-attention network (ArecaNet) to reduce information loss and fuse extracted features from sub-networks in an FER system. The proposed ArecaNet consists of the steps of extracting channel and spatial features from the main network, extracting landmark features from the sub-networks, fusion of the features of the main network and sub-networks into the assembled residual enhanced cross-attention network, and recognizing emotions through the fused features. The experimental results show that the proposed ArecaNet achieves 97.0% and 97.8% accuracy for the public DBs FER-2013 and RAF-DB, respectively, and is 4.5% better than the existing state-of-the-art (SOTA) model PAtt-Lite for FER-2013 and 2.75% better for RAF-DB. ArecaNet has solved the overfitting limitation of a single network and the information loss limitation of features extracted from multiple networks by improving the recognition accuracy in the test environment. Our method addresses the issue of information loss commonly observed in traditional ensemble approaches by introducing an enhanced cross-attention mechanism. This novel design enables the seamless and efficient integration of information across diverse networks, ensuring that complementary features are effectively preserved and utilized.

The paper is structured as follows: Section 1 introduces the background, motivation, and objectives of this study. Section 2 reviews the related work, providing a comprehensive overview of the existing approaches and identifying research limitations. Section 3 describes the proposed model’s architecture and methodology in detail, including its key components and innovations. Section 4 presents the results of both comparative experiments and our self-conducted experiments, followed by an in-depth analysis of these findings. Finally, Section 5 concludes the paper with a summary of the study, highlighting the key contributions and offering insights into future research directions.

## 2. Literature Review: Related Works

**Figure 1:**
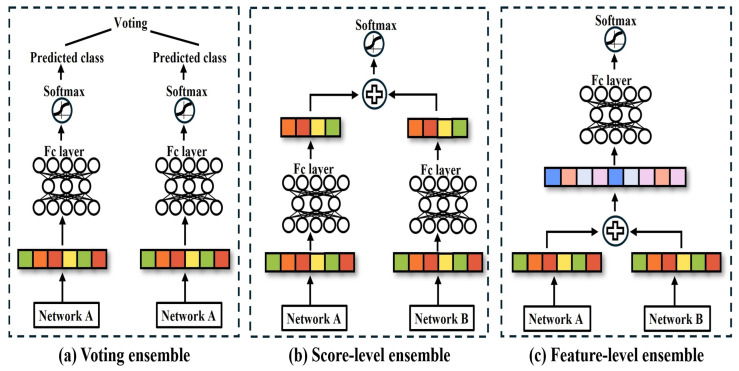
Network ensemble method: (**a**) represents the voting-based ensemble, (**b**) represents the score-level-based ensemble, (**c**) represents the feature-level-based ensemble.

Based on the research highlighted in Section 1, this section examines both single-network and ensemble-based approaches to explore the limitations and potential improvements of existing FER systems. FER systems play a crucial role in advancing HCI and HRI technologies. However, CNN- and ViT-based single networks suffer from overfitting to the training database, resulting in a low classification accuracy in real-world environments. Research related to this issue is discussed in Section 2.1, where the characteristics and performance of single-network-based approaches are analyzed, and their major limitations are identified. Section 2.2 focuses on existing ensemble-based methods proposed to overcome the shortcomings of single networks. As illustrated in Figure 1, ensemble methods include (a) voting ensembles, (b) score-level ensembles, (c) feature-level ensembles, which are each analyzed for their structural characteristics and performance differences.

Both single-network and ensemble-based approaches exhibit specific structural and performance limitations. This section conducts an in-depth analysis of these methodologies to identify areas for improvement, forming the foundation for the model design proposed in Section 3.

### 2.1. Single Head Approach for FER

As the categories of images used in an FER system are broad, such as race, gender, age, expression, pose, and image quality, there is a limit to improving the accuracy of emotion recognition. Since the features extracted from the network determine the emotion recognition performance, feature exchange, fusion, and lossless feature extraction are important. With the increase in GPU and computing power and the development of deep neural networks, the analysis of complex nonlinear patterns in high-dimensional space has become possible through feature extraction methods based on convolution operations. CNNs can extract high-dimensional nonlinear features that were impossible to extract through HOG and LBP; but, the filter-based convolution operation has a limitation in that it is limited to local feature extraction. ViT can solve the limitation of CNN’s local feature extraction with the attention mechanism and analyze the correlation between global features and the importance of features. A single-network study using CNN and ViT is being conducted to expand the model size, such as the number of layers, and improve the performance by changing the structure. Minaee et al. [14] used the method of Jaderberg et al. [15] to weight the eyes, nose, and mouth of a face using a spatial transformer and shallow CNN network. Sekaran et al. [16] recognized emotions by optimizing AlexNet pretrained with ImageNet through fine-tuning using FER-2013 DB. Riaz et al. [17] separated feature extraction into three stages and used the feature extraction method of the parallel network layer from Inception [18] in the intermediate feature extraction process. Zhao et al. [19] proposed a lightweight network for an FER system using ShuffleNet-V2 [20], and extracted global and local features simultaneously using a channel spatial modulator along with local feature extraction. Kim et al. [21] extracted robust features while reducing the feature dimension using the squeeze module from local and global features of a face image, and designed it by considering the correlation between the extracted features using ViT. Xue et al. [22] proposed a method to remove the unimportant parts in emotion classification and focus on the important features by combining the attentive patch pooling (APP) module and the attentive token pooling (ATP) module with ViT. Although various methodologies have been proposed to extract local and global features, it is difficult to improve performance in real environments with a single network due to the problems of overfitting and noise included in the training DB. Ensemble-based studies that are robust to overfitting and noise are being conducted to improve performance in real environments.

### 2.2. Ensemble-Based Approach for FER

Ensemble-based network design is being studied to improve overfitting and noise in a single network, as shown in Table 1. Ensemble-based networks are divided into voting-based ensemble, score-level ensemble, and feature-level ensemble methods. Voting-based ensemble applies majority or weighted voting to the prediction results of individual networks to determine the final class. Score-level ensemble combines the scores of features extracted from each network to make the final prediction, and adds or averages the output scores to output the final logits. Feature-level ensemble concatenates the features of the same dimensionality extracted from each network to make the final prediction, and uses the combined feature vector as the input to make a prediction through the final classifier.

Shirsath et al. [23] achieved a 67% accuracy for the FER-2013 DB using the Xception CNN architecture and max voting ensemble method. Chang et al. [24] combined four pre-trained models, VGG-19, VGGFace, ViT-B/16, and ViT-B/32, using a hard voting (majority voting) method and achieved a 76.30% accuracy for the FER-2013 DB. Yu et al. [25] combined three face detectors and used an ensemble of multiple CNN models to achieve a 61.29% accuracy for the SFEW DB through a score-level ensemble. Lahariya et al. [26] used an ensemble with two CNN models (Mini-Xception and a four-layer CNN model) and fused them at the score level through an average layer method and achieved a 68% accuracy for the FER-2013 DB.

Karnati et al. [13] extracted facial edge information as (M, D) using a local gravitational force (GF) descriptor and classified seven emotions using a score-level ensemble using the parallel learning of a local and holistic DCNN, and achieved about 78% accuracy for FER2013 and about 83% accuracy for RAF-DB. Karnati et al. [30] applied residual dilated multi-scale (RDMS) and spatial/channel attention to a blended feature attention network (BFAN) to correct illumination deviation, segment the face into five regions, alleviate pose and occlusion problems, and prevent overfitting, and used a score-level-based ensemble method. The developed model achieved about 84.89% accuracy for FER2013 and about 90.86% accuracy RAF-DB. Yu et al. [27] combined the features of facial images with three networks, ViT, MANet, and ResNet, into a feature-level ensemble and achieved an 80.86% accuracy for RAF-DB(CE). Georgescu et al. [28] combined the facial features extracted from VGG-13, VGG-f, and VGG-face with the facial features computed by the bag-of-visual-words model into a feature-level ensemble and achieved an 87.76% accuracy for FER+ DB. Liu et al. [29] combined the pixel-level features of facial images extracted by SACNN and the geometric-level features of facial landmarks in different facial regions extracted by ALSTMs into a feature-level ensemble and achieved a 74.31% accuracy for FER-2013 DB.

They conducted research on improving accuracy in real test environments through ensemble methods. However, their findings indicate that traditional ensemble approaches still face limitations in achieving substantial improvements in accuracy beyond specific thresholds. Existing ensemble methods proceed with the operation process of voting-based, score-based, and embedding vector combination-based ensembles. Existing ensemble methods have limitations in single-network dependency and information loss in the ensemble operation process. It is necessary to study methods for minimizing information loss in the ensemble process for the fusion of features extracted from each network. Therefore, it is essential to develop new ensemble methods that minimize information loss during the fusion of features extracted from each network. This study aims to address these limitations by proposing ArecaNet, an assembled ensemble architecture that integrates a main network and two complementary sub-networks through residual enhanced cross-attention (RECA). Unlike conventional voting-based or concatenation-based ensemble strategies that suffer from information loss and limited interaction between networks, the proposed RECA mechanism enables effective feature exchange while preserving the original representations through its residual design. By reducing single-network dependency and minimizing information degradation during fusion, ArecaNet achieves more discriminative feature integration for robust FER performance. The following section describes the detailed architecture and operational principles of the proposed model.

## 3. Assembled Residual Enhanced Cross-Attention Networks for Robust Emotion Recognition

**Figure 2:**
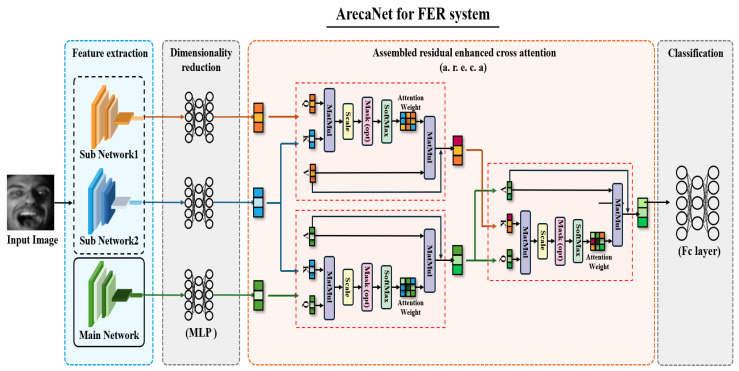
Overall architecture of ArecaNet model for FER system.

Existing ensemble networks exhibit a performance determined by a single network, or important information exchange between networks is not sufficient and the feature information of each network is lost during the ensemble process. This study proposes ArecaNet using assembled residual enhanced cross-attention to reduce the loss of information extracted from sub-networks and to fuse the extracted features. ArecaNet consists of the process of extracting channel and spatial features from the main network, the process of extracting landmark features from the sub-network, the process of fusion of the features of the main network and sub-network using assembled residual enhanced cross-attention, and the process of recognizing emotions through the fused features, as shown in Figure 2.

The ArecaNet introduced in this paper gradually absorbs and fuses the information extracted from the sub-networks (MobileFaceNet, ir50) through the residual enhanced cross-attention (r.e.c.a) process with the value vector extracted from the main network: spatial and channel squeeze and excitation shallow resnet (SCSESResNet). The knowledge distillation procedure is included so that the main network repeatedly receives the feature information of the sub-networks. The r.e.c.a process in the form of knowledge distillation reduces the risk of a single network dominating the overall performance and also alleviates the problem of redundant features being combined, ultimately minimizing information loss. Finally, the important features of the facial image extracted through the attention operation within the r.e.c.a process are not damaged and are reflected in the final logit calculation, which is different from the existing score-level ensemble, feature-level ensemble, and single cross-attention methods.

Feature extraction involves sub-network 1, represented by MobileFaceNet [31], and sub-network 2, which is the ir50 [32] network pre-trained on the Ms-Celeb-1M [33] database and utilized in POSTER++ [34]. The main network is designed as a spatial and channel squeeze and excitation attention shallow ResNet (SCSESResNet), applying scSE attention [35] to ResNet [36]. For dimensionality reduction, MLP (multi-layer perceptron) is employed. In the third stage, assembled residual enhanced cross-attention, r.e.c.a stands for residual enhanced cross-attention. Lastly, the FC layer, acting as the classification head, serves as the final fully connected layer.

### 3.1. Comparison of Attention Mechanisms

**Figure 3:**
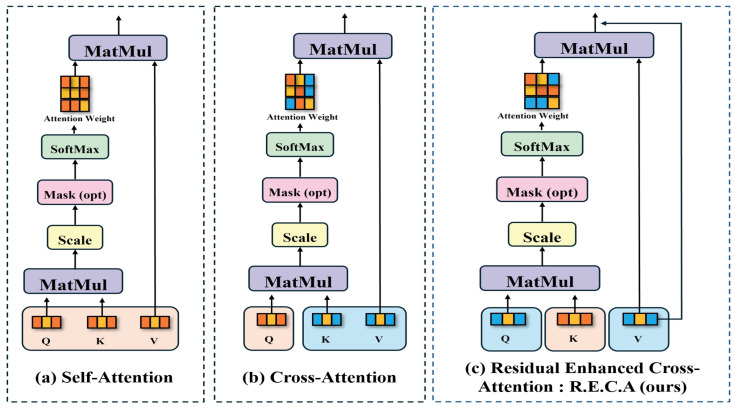
Analysis of existing attention methods and the proposed attention method: (**a**) represents self-attention, (**b**) represents cross-attention, (**c**) represents residual enhanced cross-attention.

Figure 3a shows the self-attention method proposed by Vaswani et al. [37], Figure 3b shows the cross-attention proposed by Chen et al. [7], and Figure 3c shows the residual enhanced cross-attention proposed by ArecaNet.

Equation (1) describes the self-attention operation process, where *Q* is the query vector, *K* is the key vector, *V* is the value vector, and $d_{q}$ denotes the dimension of the key vector. *Q*, *K*, and *V* in Equation (1) stand for the query, key, and value vectors extracted from the same network. *Q*, $K_{*}$, and $V_{*}$ in Equation (2) indicate that *Q* and $\left(K_{*},V_{*}\right)$ are vectors extracted from different networks of the same image, and $Q_{*}$, *K*, and $V_{*}$ in Equation (3) show that *K* and $\left(Q_{*},V_{*}\right)$ are vectors extracted from different networks. In each process, the inner product is calculated using $QK^{⊤}$ with the defined query, key, and value vectors. The attention weight is calculated by applying the softmax function to the calculated inner product value. The calculated weight is used to find important parts in the value vector. As a result, the attention weights are weighted and added to the value vector to output the final attention result. In the cross-attention process of Equation (2), the query and key are composed of different network feature vectors, unlike the generic attention of Equation (1), and the information between different feature vectors is fused. Equation (3) is composed of *K* and $\left(Q_{*},V_{*}\right)$ pairs instead of *Q* and $\left(K_{*},V_{*}\right)$ pairs in Equation (2). The *K* and $\left(Q_{*},V_{*}\right)$ pairs composed in the calculation of Equation (3) ultimately extract $V_{*}$ vectors and question vector $Q_{*}$ from the same network. The weights for the question vector $Q_{*}$ are output from the *K* vector extracted from another network, and the $V_{*}$ vector is used as the subject that receives information. The query and key composition of Equation (3) enables fusion while preserving information more efficiently than Equation (2). The residual operation process can reduce the information loss of $V_{*}$ by adding the original $V_{*}$ vector to the $V_{*}$ vector that has been given attention weights.

(1) $$\mathrm{Self-Attention}\;\left(Q,K,V\right)=\mathrm{softmax}\left(\frac{QK^{⊤}}{\sqrt{d_{q}}}\right)V$$

(2) $$\mathrm{Cross-Attention}\;\left(Q,K^{*},V^{*}\right)=\mathrm{softmax}\left(\frac{QK^{*⊤}}{\sqrt{d_{q}}}\right)V^{*}$$

(3) $$R.E.C.A\left(Q^{*},K,V^{*}\right)=\left(\mathrm{softmax}\left(\frac{Q^{*}K^{⊤}}{\sqrt{d_{q}}}\right)V^{*}\right)+V^{*}$$

### 3.2. Assembled Residual Enhanced Cross-Attention Networks

Figure 2 shows the overall structure of ArecaNet. ArecaNet extracts features using one main network and two sub-networks and recognizes emotions using the features extracted from each network. The main network uses scSE attention shallow ResNet [36] which reduces the number of convolutional blocks consisting of two layers at each stage of ResNet18 to one layer and applies scSE Attention [35] to each block process to extract spatial and channel features. The sub-networks use ir50 [32] for extracting overall features of the face and MobileFaceNet [31] for extracting landmark features of the face. The features extracted from each network are $F_{\mathrm{Sub}1},\;F_{\mathrm{Sub}2},\;F_{\mathrm{Main}}\in R^{B\times H\times W\times C}$, where *B* is the batch size, *H* and *W* are the height and width of the input image, and *C* is the number of channels. These features are calculated using Equation (4). In Equation (5), the average value of the features extracted with a fixed kernel size for the features in the form of $R^{B\times H\times W\times C}$ through average pooling is calculated to reduce the spatial resolution and change it to $R^{B\times H^{\prime }\times W^{\prime }\times C}$. The calculation of Equation (5) improves the generalization ability, plays the role of reinforcing position invariance, and reduces the spatial resolution. The features extracted from each network are fused through the r.e.c.a process, as shown in Figure 3. The feature image is converted into a one-dimensional vector in the form of $R^{B\times H^{\prime }\cdot W^{\prime }\cdot C}$ through the calculation of Equation (6). In order to reduce the amount of computation in the r.e.c.a process, the dimension of the one-dimensional vector is reduced through the calculation of Equation (7). In the r.e.c.a module, the reduced features from sub-network 1 and sub-network 2 are first integrated to form a combined representation, which is used as the key vector. The reduced feature of the main network serves as both the query and value. Through Equation (3), the main network attends to the complementary information provided by the combined sub-network features, producing an updated feature representation. A residual connection is then applied to preserve the original characteristics of each network while enhancing discriminative information. The final output of the a.r.e.c.a block, shown in Figure 2, is a refined feature vector that retains important information from all networks with minimized loss, providing a more robust representation for emotion recognition.

(4) $$\begin{array}{cc} F_{\mathrm{Sub}1} & =\mathrm{MobileFaceNet}(X)\\ F_{\mathrm{Sub}2} & =\mathrm{ir}50(X)\\ F_{\mathrm{Main}} & =\mathrm{SCSESResNet}(X) \end{array}$$

Here, *F* denotes the features extracted from the input image *X* by each network. Since we designate MobileFaceNet and ir50 as sub-network 1 and sub-network 2, respectively, and SCSESResNet as the main network, we use the notations $F_{\mathrm{Sub}1}$, $F_{\mathrm{Sub}2}$, and $F_{\mathrm{Main}}$ to represent the features extracted by these respective networks.

(5) $$F_{\mathrm{Sub}1}^{\prime },\;F_{\mathrm{Sub}2}^{\prime },\;F_{\mathrm{Main}}^{\prime }=\mathrm{AvgPool}\left(F_{\mathrm{Sub}1},\;F_{\mathrm{Sub}2},\;F_{\mathrm{Main}}\right)$$

When $F_{\mathrm{Sub}1},\;F_{\mathrm{Sub}2}$, and $F_{\mathrm{Main}}$ are passed through average pooling, the spatial resolution of the features is reduced, altering the height (*H*) and width (*W*) dimensions. Consequently, we denote the output features as $F_{\mathrm{Sub}1}^{\prime },F_{\mathrm{Sub}2}^{\prime },$ and $F_{\mathrm{Main}}^{\prime }$ to indicate that they have been processed by the average pooling operation.

(6) $$F_{\mathrm{Sub}1}^{R},F_{\mathrm{Sub}2}^{R},F_{\mathrm{Main}}^{R}=\mathrm{Reshape}\left(F_{\mathrm{Sub}1}^{\prime },F_{\mathrm{Sub}2}^{\prime },F_{\mathrm{Main}}^{\prime }\right)$$

The process of flattening the features obtained after average pooling into one-dimensional vectors is defined as a reshape operation. The notation *R* added to *F* ($F_{\mathrm{Sub}1}^{R},F_{\mathrm{Sub}2}^{R},F_{\mathrm{Main}}^{R}$) signifies that the features $F_{\mathrm{Sub}1}^{\prime },F_{\mathrm{Sub}2}^{\prime },$ and $F_{\mathrm{Main}}^{\prime }$ have been transformed into one-dimensional flattened vector representations.

(7) $$F_{\mathrm{Sub}1}^{\mathrm{out}},F_{\mathrm{Sub}2}^{\mathrm{out}},F_{\mathrm{Main}}^{\mathrm{out}}=\mathrm{MLP}\left(F_{\mathrm{Sub}1}^{R},F_{\mathrm{Sub}2}^{R},F_{\mathrm{Main}}^{R}\right)$$

An MLP refers to a multi-layer perceptron, which is applied to the one-dimensional flattened features to perform dimensionality reduction. After passing through the MLP, the reduced one-dimensional feature vectors are denoted using the out notation ($F_{\mathrm{Sub}1}^{\mathrm{out}},F_{\mathrm{Sub}2}^{\mathrm{out}},F_{\mathrm{Main}}^{\mathrm{out}}$), representing the final compressed features.

## 4. Experimental Studies

### 4.1. Experiment Environments

The proposed ArecaNet was designed using the PyTorch 2.7.0 library, and the recognition performance was analyzed using the public FER-2013 and RAF-DB datasets in the NVIDIA TESLA V100 GPU environment on the Colab platform. Both FER-2013 and RAF-DB were collected under unconstrained imaging conditions using standard camera sensors, which inherently introduce variations such as illumination changes, pose differences, occlusion, and resolution imbalance. These sensor-induced variations reflect real-world sensing environments, and ArecaNet is evaluated under these conditions to verify its robustness to such sensing-related factors. The FER-2013 DB [38] consists of 28,709 training data images and 3589 test data images, and the RAF-DB [39] consists of 12,271 training data images and 3068 test data images. The FER-2013 and RAF DBs contain the same seven emotions: angry, disgust, fear, happy, sad, surprise, and neutral. The study of the seven emotion recognition performances makes an important contribution to improving the recognition performance of the network and enhancing its reliability in real environments.

ArecaNet uses the ir50 [32] network pre-trained on the Ms-Celeb-1M DB [33] used in POSTER++ [34] and MobileFaceNet [31] as sub-networks for facial landmark detection, and the main network is designed as scse attention shallow resnet (SCSESResNet) that applies scSE attention [35] to ResNet [36]. During the training, augmented face images were used by applying RandomHorizontalFlip, RandomAdjustSharpness, ColorJitter, and Random cropping. The hyperparameters for learning were set to 100 epochs, 32 batch size, $1\times 10^{-3}$ learning rate, $1\times 10^{-3}$ weights, and $1\times 10^{-4}$ decay in the AdamW optimizer [40]. The loss function was a standard cross entropy loss, and the recognition system for seven emotional images of FER-2013 and RAF-DB was compared and evaluated with existing methods.

The emotion recognition system is evaluated by true positive (TP), false positive (FP), false negative (FN), and true negative (TN). TP is the case where the system correctly recognizes the correct emotion. FP is the case where the system recognizes an emotion that is not actually correct as an incorrect answer and misclassifies it. FN is the case where the system processes the correct emotion as an incorrect answer and fails to recognize the emotion. TN is the case where the system correctly recognizes a state that is not correct and processes it as an incorrect answer. The accuracy (Acc) in Equation (8) represents the proportion of correct predictions made by the system, where higher counts of true positives (TP) and true negatives (TN) lead to a higher accuracy. In Equation (8), TP, TN, FP, and FN denote the numbers of true positives, true negatives, false positives, and false negatives, respectively. The F1 score in Equation (9) is the harmonic mean of precision and recall, and is an indicator to evaluate the prediction performance of the model in an imbalanced DB. The higher the F1 score, the more accurately and consistently the model predicts the positive class.

(8) $$\mathrm{Accuracy}=\frac{\mathrm{TP}+\mathrm{TN}}{\mathrm{TP}+\mathrm{TN}+\mathrm{FP}+\mathrm{FN}}$$

(9) $$F1\;\mathrm{Score}=\frac{2\times \mathrm{TP}}{2\times \mathrm{TP}+\mathrm{FP}+\mathrm{FN}}$$

### 4.2. Ablation-CAM Results for Each Network in ArecaNet

**Figure 4:**
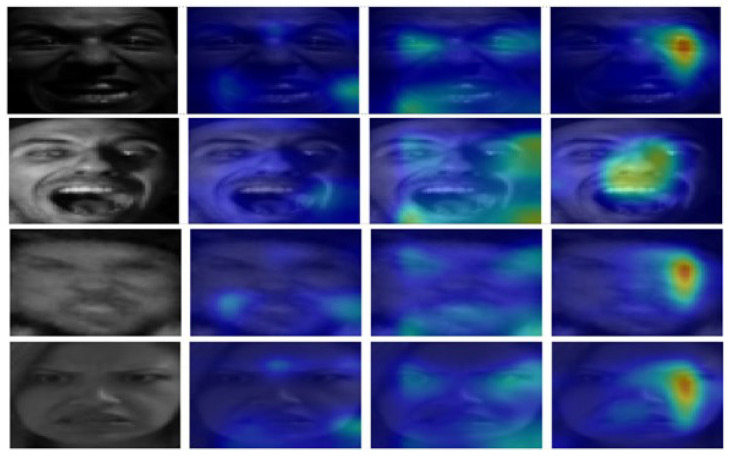
Original input image (first column), Ablation-CAM through ResNet + scSE (second column), Ablation-CAM through ir50 (third column), and Ablation-CAM through MobileFaceNet (fourth column). It can be observed that the final features extracted from each network focus on different parts of the image for class prediction.

Figure 4 shows the Ablation-CAM results of SCSESResNet, which is the main network of ArecaNet, ResNet, and its sub-networks, ir50 and MobileFaceNet, which applied scSE attention to the main networks of ArecaNet and ResNet, for less than 10 epochs. In the early stage of training, each network extracted facial features by focusing on different parts of the same facial image. While the main network and ir50 observed relatively omnidirectional features of the facial image, MobileFaceNet focused on a specific part. The Ablation-CAM results for each network for the same image show that the features extracted from each network were different before applying the assembled residual enhanced cross-attention. The emotion recognition accuracies of 97.0% and 97.8% in FER-2013 and RAF-DB prove that ArecaNet minimized information loss and effectively fused the features extracted from each network.

### 4.3. Comparative Analysis Between the Conventional Ensemble Methods and ArecaNet

Table 2 shows the results of comparing the emotion recognition accuracy of the assembled residual enhanced cross-attention-based ensemble proposed in ArecaNet and the existing score-level and feature-level ensembles, which are fusion methods, for each feature extracted from the main network and sub-network of ArecaNet. For RAF-DB, ArecaNet was verified to be 16.1% more accurate than the score-level ensemble and 15.7% more accurate than the feature-level ensemble. For FER-2013, ArecaNet was verified to be 26.12% more accurate than the score-level-based fusion and 25.9% more accurate than the feature-level-based fusion, and ArecaNet’s feature fusion method was evaluated to be superior to the existing ensemble method.

**Figure 5:**
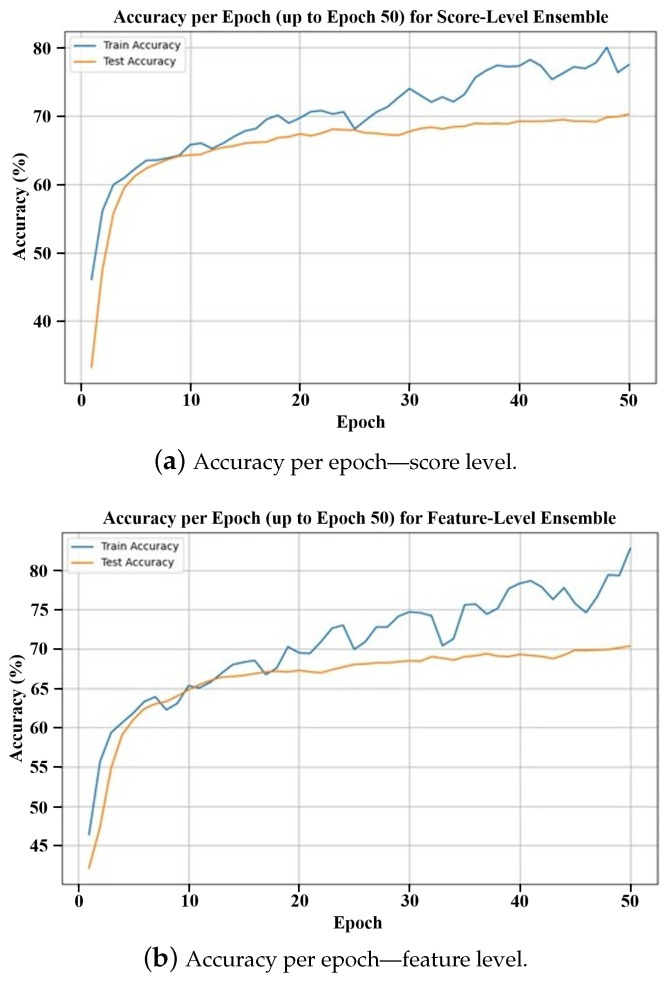

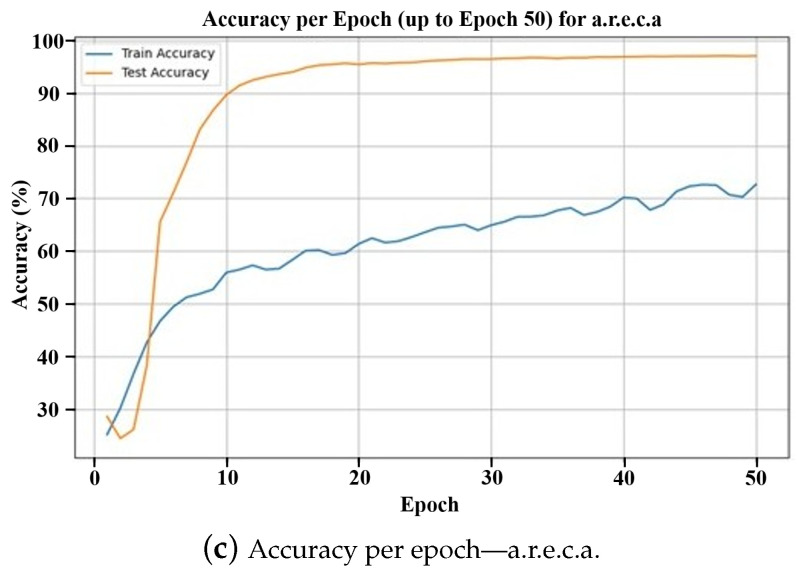
Accuracy comparison over 50 epochs with the FER-2013 dataset for the three ensemble strategies.

Figure 5 is a graph comparing the accuracy of the score-level ensemble and feature-level ensemble methods and the proposed ArecaNet during 50 epochs of training and testing on the FER-2013 DB. The experiment in Figure 5 was conducted to measure how many training processes are needed for high emotion recognition accuracy in the test environment. The emotion recognition accuracy was evaluated using the training and test datasets for each epoch. In Figure 5a,b, the score-level and feature-level ensembles show the accuracy improvement limit and overfitting phenomenon as the accuracy converges to 70.2% and 70.35%, respectively, in the test environment during 50 epochs. ArecaNet achieves a 65.56% test accuracy after only 5 epochs of training and 89.57% after 10 epochs in Figure 5c. ArecaNet performed pattern recognition for emotion recognition more accurately and efficiently with less training time than existing ensemble methods such as score-level and feature-level. Some signs of overfitting appear in ArecaNet. While the score-level ensemble and feature-level ensemble converge at around 70% in test accuracy, ArecaNet is analyzed as having an additional accuracy performance improvement even after the accuracy converges at around 95%. Since the test accuracy at the convergence point is different, ArecaNet cannot be interpreted as having the same level of overfitting as the score-level and feature-level ensembles. The test accuracy of over 90% in 10 epochs in ArecaNet is achieved due to its high generalization performance.

### 4.4. Performance Metrics Comparison Between SOTA Methods and ArecaNet

**Figure 6:**
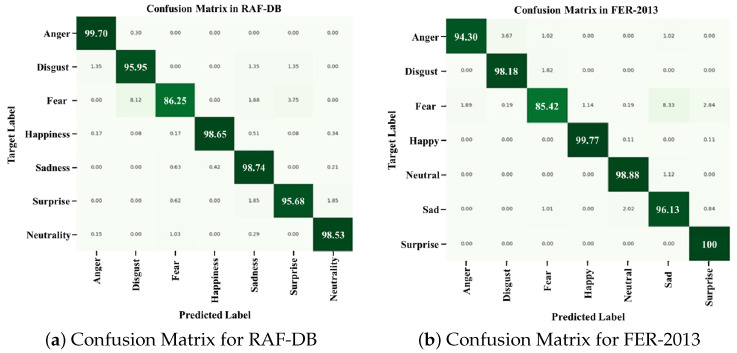
Confusion matrices for RAF-DB and FER-2013 with ArecaNet.

Table 3, Table 4 and Table 5 and Figure 6 show the comparative analysis of the performance of ArecaNet with existing SOTA methods using the FER-2013 and RAF-DB databases. Table 3 shows the comparison of accuracy by existing method, Table 4 shows the comparison of accuracy for emotion by existing method, and Table 5 shows the results of the comparative analysis using the SOTA model and F1 score. Figure 6 shows the confusion matrices of ArecaNet in each DB.

Table 3 shows the results of a comparative analysis of the performance of ArecaNet with existing SOTA methods. FER-2013 has limited improvement in recognition performance due to label error and face missing problems. For FER-2013, refs. [41,42,43,44,45,46] achieved accuracies of 73.73%, 74.94%, 71.83%, 73.45%, 74.77%, and 71.44%, showing limited improvements in accuracy. The SOTA model PAtt-Lite [48] showed a high improvement in accuracy with 92.5% for FER-2013, but the proposed ArecaNet was verified to have 97.0% accuracy, which is 4.5% higher than PAtt-Lite. RAF-DB also has limited improvement in recognition performance due to various ages, genders, races, and poses. For RAF-DB, ArecaNet was validated to be 97.8% accurate, outperforming Qi et al. [41] by 10.28%, Yang et al. [46] by 6.89%, Mao et al. [34] by 5.59%, and PAtt-Lite [48] by 2.75%, which achieved the SOTA on RAF-DB. As a result, we confirmed that ArecaNet achieved a new SOTA accuracy for FER-2013 and RAF-DB.

Table 4 shows the results of comparing the emotion recognition accuracy of each SOTA method and ArecaNet for each emotion class in RAF-DB. Figure 6 shows the class-specific detailed performance using ArecaNet with RAF-DB and FER-2013 through a confusion matrix. In Table 4, the disgust and fear emotions show a limitation of performance improvement through recognition accuracy in all studies [34,44,47,48,49] due to low accuracy. For the disgust emotion, ArecaNet was evaluated to have an accuracy of 95.95%, which was 35.95% higher than [49], which recorded the lowest accuracy, and 15.95% higher than the SOTA model PAtt-Lite [48]. For the fear emotion, where the improvement in accuracy is limited compared with the disgust emotion, ArecaNet was evaluated to have an accuracy of 86.25%, which is 25.25% higher than [44,49], which recorded the lowest accuracy, and 13.28% higher than the SOTA model PAtt-Lite [48]. ArecaNet was also analyzed to have a high accuracy in classes where the improvement in the accuracy of disgust and fear emotion recognition was limited. For the happiness emotion, which was recognized with a high accuracy by all existing methods, ArecaNet was evaluated to have an accuracy of 98.65%, which is 4.65% higher than [49], which recorded the lowest accuracy, and 0.68% higher than the SOTA model PAtt-Lite [48]. In all emotions except for the surprise emotion and in the overall accuracy, ArecaNet achieved a higher accuracy than the SOTA model PAtt-Lite [48]. In Table 5, ArecaNet was verified to be superior to the SOTA model PAtt-lite in all emotion recognition accuracies as well as F1 score, proving the robustness of the network in data imbalance situations.

### 4.5. ArecaNet Ablation Analysis

Table 6 shows the results of comparing and analyzing the accuracy differences according to the ablation stage for each network module that constitutes ArecaNet in the RAF-DB DB. In Table 6, ‘w/o’ denotes ‘without,’ indicating the removal of the corresponding module. The ablation study removed the corresponding modules from the entire model and retrained them for a total of 150 epochs from epoch 1 under the same hyperparameters such as learning rate and batch size. The additional ablation study did not use learning methods such as freezing weights and partial removal for a reliable experimental environment. When the r.e.c.a process of Figure 3 is performed only once using the sub-network ir50 of ArecaNet and the main network, the accuracy is evaluated as 95.5%, which is 2.3% lower than ArecaNet with 97.8% accuracy. When the r.e.c.a process is performed only once using the sub-network MobileFaceNet and the main network, the accuracy is evaluated as 95.9%, which is 1.9% lower than ArecaNet with 97.8% accuracy. When both ir50 and MobileFaceNet are removed and only the main network is used, the accuracy is evaluated as 80.4%, which is 17.4% lower than ArecaNet with 97.8% accuracy. All the decreases in emotion recognition accuracy after removing the sub-networks of ArecaNet show that each network contributes to different important feature extraction in the recognition process. As a result, it was proven that all the sub-network features and the features extracted through r.e.c.a are more accurate feature information for emotion recognition than the features extracted through a single r.e.c.a process or when not operated through r.e.c.a. When cross-attention in Figure 3b is used to replace r.e.c.a in Figure 3c , the accuracy was evaluated to be 88.1%, which was 9.7% lower than ArecaNet, with an accuracy of 97.8%. The 9.7% performance improvement when replacing cross-attention with r.e.c.a shows that the information on the query and value vectors, which are feature vectors of the main network, is effectively extracted and fused from the key vectors of the sub-network.

## 5. Conclusions

HCI technology utilizing human biometric information with the development of artificial intelligence and robotics is being actively studied. The FER system is being applied in various fields such as personalized healthcare, education, and recommendation services by integrating BER and SER. The existing FER system has limitations in improving emotion recognition accuracy because facial images collected through camera sensors often contain real-world noise such as illumination variations, occlusion, pose differences, and resolution imbalance which further exacerbate overfitting and degrade feature extraction when relying on a single network. To improve the recognition accuracy, different network features are integrated using ensemble techniques such as voting ensemble, score-level ensemble, and feature-level ensemble methods. The existing ensemble method has a performance determined by a single network, or important information exchange between networks is not sufficient and the feature information of each network is lost during the ensemble process.

This paper proposes ArecaNet, which applies assembled residual enhanced cross-attention to robustly analyze faces, in order to solve the overfitting problem of a single network and the information loss problem in the existing ensemble process. The proposed ArecaNet consists of the process of extracting channel and spatial features from the main network, the process of extracting landmark features from the sub-network, the process of fusing the features of the main network and the sub-network with assembled residual enhanced cross-attention, and the process of recognizing emotions through the fused features. The experimental results show that ArecaNet is 4.8% more accurate for FER-2013 and 2.8% more accurate for RAF-DB than PAtt-Lite, which achieved the SOTA performance for FER-2013 and RAF-DB, and achieved a new SOTA performance for each DB. We verified that ArecaNet maximizes the exchange of features extracted from the main network and the sub-network to select important features of facial images and ultimately improves the emotion recognition performance. While ArecaNet uses 24.95 M parameters and 6.57 G FLOPs, the network model developed by J. Le Ngwe et al. [48] is very lightweight, with about 1.1 M parameters. The network model developed by M. Karnati [30] is lightweight, with 9.18 M parameters and 0.49 G FLOPs, but achieves a high accuracy. For future research, we plan to conduct research on lightweighting for application in real environments like the models developed in both studies, and on other modalities such as BER and SER and information fusion processes.

## Figures and Tables

**Table 1:** Comparative analysis of accuracy across various studies in ensemble methods.

Ensemble Method	Network	DBs	Accuracy (%)	Author
Voting	Trained Xceptions	FER-2013	67.0	S. Shirsath et al. [23]
	VGG-19, VGGFace, ViT-B/16, ViT-B/32	FER-2013	76.3	J. X. Chang et al. [24]
Score level	Multiple Basic CNN	SFEW	61.3	Z. Yu et al. [25]
	Mini-Xception, 4layerCNN	FER-2013	68.0	A. Lahariya et al. [26]
Feature level	Vit, MANet, ResNet	RAF-DB (CE)	80.9	J. Yu et al. [27]
	VGG-13, VGG-f, VGGFace, BOVW	FER+	87.8	M. I. Georgescu et al. [28]
	SACNN, ALSTMs	FER-2013	74.3	C. Liu et al. [29]

**Table 2:** Comparative accuracy of proposed and existing ensemble methods for RAF-DB and FER-2013.

DB	Score Level Ensemble	Feature Level Ensemble	ArecaNet (Ours)
RAF-DB	81.7	82.1	97.8
FER-2013	70.9	71.1	97.0

**Table 3:** Accuracy-based comparison of proposed and existing methods for FER-2013 and RAF-DB.

Methods	FER-2013	RAF-DB
PCARNet [41]	73.73	87.52
Ensembled-T Net [42]	74.94	88.17
RUL-C [43]	71.83	89.51
ResNet18+IRAM [44]	73.45	89.73
GCNN [45]	74.77	89.87
RASN [46]	71.44	90.91
POSTER [47]	-	92.05
POSTER++ [34]	-	92.21
PAtt-Lite [48]	92.50	95.05
ArecaNet (ours)	97.0	97.8

**Table 4:** Per-class performance evaluation of proposed and existing methods for RAF-DB. The highest accuracy for each class is highlighted in bold, with the second-highest underlined.

Methods	Anger	Disgust	Fear	Happiness	Sadness	Surprise	Neutrality	Mean 7-Class
Ad-Corre [49]	79	60	61	94	83	88	88	79.01
ResNet18+IRAM [44]	85	64	61	96	91	86	91	82
POSTER++ [34]	88.27	71.88	68.92	97.22	92.89	90.58	92.06	85.97
POSTER [47]	88.89	75.00	67.57	96.96	91.21	90.27	92.35	86.04
PAtt-Lite [48]	95.68	80.00	72.97	97.97	93.93	**96.05**	96.03	90.38
ArecaNet (ours)	**99.70**	**95.95**	**86.25**	**98.65**	**98.74**	95.68	**98.53**	**96.21**

**Table 5:** Comparison of F1 scores between the proposed model and previous SOTA models for RAF-DB and FER-2013. Note that the F1 score for the PAtt-Lite model was calculated based on its confusion matrix, as the original paper does not provide a separate F1 score.

Dataset	Anger	Disgust	Fear	Happiness	Sadness	Surprise	Neutrality
RAF-DB							
PAtt-Lite [48]	0.964	0.858	0.828	0.979	0.880	0.928	0.887
ArecaNet (ours)	0.990	0.939	0.919	0.986	0.966	0.951	0.981
Improvement	+2.7%	+9.4%	+10.99%	+0.72%	+9.77%	+2.48%	+10.6%
FER-2013							
PAtt-Lite [48]	0.777	0.873	0.830	0.986	0.931	0.974	0.969
ArecaNet (ours)	0.961	0.972	0.903	0.993	0.931	0.981	0.983
Improvement	+23.68%	+11.34%	+8.8%	+0.71%	+0.0%	+0.72%	+1.44%

**Table 6:** Ablation analysis: ArecaNet accuracy performance comparison for RAF-DB.

Method	Accuracy (%)
ArecaNet	97.8
w/o ir50	95.5
w/o MobileFaceNet	95.9
w/o ir50 & MobileFaceNet	80.4
w/o a.r.e.c.a (with cross-attention)	88.1

## Data Availability

No new data were generated. Experiments used two public datasets: (i) FER-2013 (CC0 licence) available at https://www.kaggle.com/datasets/msambare/fer2013 (accessed on 1 October 2025); (ii) RAF-DB, obtainable for academic research from the original authors at http://www.whdeng.cn/RAF/model1.html (accessed on 1 October 2025).
